# Medical Students’ Perceptions of Play and Learning: Qualitative Study With Focus Groups and Thematic Analysis

**DOI:** 10.2196/25637

**Published:** 2021-07-28

**Authors:** A E J Van Gaalen, A D C Jaarsma, J R Georgiadis

**Affiliations:** 1 Department of Biomedical Sciences of Cells and Systems, Section Anatomy & Medical Physiology University Medical Center Groningen University of Groningen Groningen Netherlands; 2 Center for Education Development and Research in Health Professions University Medical Center Groningen University of Groningen Groningen Netherlands

**Keywords:** gamification, serious games, game-based learning, medical education, computers, new technology, focus group, play, qualitative

## Abstract

**Background:**

In times where distance learning is becoming the norm, game-based learning (GBL) is increasingly applied to health profession education. Yet, decisions for if, when, how, and for whom GBL should be designed cannot be made on a solid empirical basis. Though the act of play seems to be intertwined with GBL, it is generally ignored in the current scientific literature.

**Objective:**

The objective of our study was to explore students’ perceptions of play in leisure time and of GBL as part of a mechanistic, bottom-up approach towards evidence-informed design and implementation of GBL in health profession education.

**Methods:**

We conducted 6 focus group discussions with medical and dentistry students, which were analyzed using thematic analysis.

**Results:**

A total of 58 students participated. We identified 4 major themes based on the students’ perception of play in leisure time and on the combination of play and learning. Our results indicate that, while play preferences were highly various in our health profession student cohort, pleasure was the common ground reported for playing. Crucially, play and the serious act of learning seemed paradoxical, indicating that the value and meaning of play are strongly context-dependent for students.

**Conclusions:**

Four key points can be constructed from our study. First, students play for pleasure. Perceptions of pleasure vary considerably among students. Second, students consider play as inefficient. Inefficiency will only be justified when it increases learning. Third, play should be balanced with the serious and only be used for difficult or tedious courses. Fourth, GBL activities should not be made compulsory for students. We provide practical implications and directions for future research.

## Introduction

In times where distance learning is becoming the norm, game-based learning (GBL) is increasingly applied to health profession education [1,2]. Yet, recent reviews in this field indicate that GBL research is still in its infancy and that robust study designs based on sound theoretical foundations or supporting scientific evidence are scarce [3-5]. Although certain trends in GBL use can be observed, there seems to be little theoretical support to clarify the effects of GBL on academic learning [4]. Most studies report on the use of GBL applications that are tailored to specific local settings [4]. Therefore, decisions about implementing game-like interventions — if, when, how, and for whom — cannot be made on a solid empirical basis. This increases the likelihood of suboptimal and even counterproductive educational design. In this study, we took a user-driven approach in an attempt to unravel key processes that could explain why and how GBL does or does not work in health profession education, therewith advancing the science and practice in this field.

GBL has been applied based on the idea that play and learning are closely intertwined [6,7]. Intriguingly, studies investigating GBL in academic settings do not seem to explicitly scrutinize, measure, or mention play [3-5,8,9]. GBL studies mainly focus on outcome measures such as learning outcome, motivation, and likability of GBL [4] but seem to dismiss play itself. This situation seems to persist despite significant scientific interest in the fundamentals of play-learning interaction [10-13]. Generally, empirical evidence supports claims that play positively influences the learning of problem solving [14-16], creativity [17,18], and self-regulatory skills [19]. Most knowledge on this play-learning interaction stems from early experimental animal research [20,21] or research on developmental psychology in children (eg, [14,22]). By comparison, play in the realm of adult learning has received very little attention, especially with regard to GBL.

The studies that came closest to research on play-learning interactions in educational contexts for adult learning were quantitative in nature and primarily used questionnaires aimed at examining experiences with already existing games or to inform the design of a specific game [23-27]. However, these studies did not investigate how play can be elicited in students or, more specifically, which type of play can benefit or support student learning, in which situations, or under which conditions. Furthermore, because of the specific study set-up or study intent, participants in such studies may have directed their answers to a specific game or game design, which does not allow for generalization of the results to other contexts or game designs. Next to quantitative studies, qualitative approaches have been employed in order to describe adult playfulness [28] or inform game design [29-33]. Findings of these studies gave insight into self-perceived reasons for adult engagement in play such as stress relief, challenge, and friendship. However, whether these needs for play in adults can also be met in combination with learning was not explored in these studies. Thus, the links between play, academic learning, and GBL remain a blind spot in the literature.

Provided that there are meaningful play-learning interactions in GBL, even when the nature of that interaction is unknown, we need to understand how to elicit play in students. But what exactly is play, how do we define play, and how do we relate play to GBL? There is no univocal answer to any of these questions, since there is considerable disagreement in the scientific literature as to what constitutes play and games [13,34]. Interestingly, and perhaps as a logical consequence of this disagreement, there *is* strong consensus that playfulness is an individual predisposition [35] and that the liking of play is dependent on personal opinions, characteristics [36-38], and context [34]. Some propositions have been made by play scholars to classify different expressions of play and distinguish play from other behaviors such as exploration [13,39]. Probably most interesting in this regard is the distinction between *paidia* (free, spontaneous, expressive, creative forms of play) and *ludus* (rule-bound play) [40]. These heuristics can be very valuable for the theoretical conceptualization of GBL, because GBL design seems to relate much more to rule-bound “ludic” play [4,26,40] than to free, creative “paidic” play. Furthermore, the strong individual character of play that has been established in the literature seems to require qualitative research approaches to understand students’ perceptions of play and academic learning, especially in relation to GBL.

In the present study, we took inspiration from play research as a first step towards a mechanistic analysis of GBL effects. We employed the qualitative method of open focus group discussions to help us gain deeper insight into medical and dentistry students’ perceptions of play and learning by exploring their ideas, interpretations, feelings, and actions [41] as well as favorable circumstances or limitations for engaging in GBL activities. Although, at this point, we do not have scientific reasons to assume that the range of opinions on play would vary significantly across students as a function of the academic level or discipline they are enrolled in, we chose to focus on medical and dental students for 2 main reasons. First, our main teaching experience as well as our research interest in GBL lie within the context of health profession education. Second, if play preferences are indeed highly individual and contextual, this would also apply to students enrolled in a particular program or discipline. In summary, in this focus group study, we explored health professions students’ perceptions of what constitutes play, play-learning interaction, and if, how, and when GBL material should be designed and implemented in health professions education to foster their learning.

## Methods

### Context

We conducted this study at the University of Groningen, University Medical Center Groningen, the Netherlands, between March 2019 and April 2019. The 6-year undergraduate medical curriculum of the University of Groningen is comprised of a 3-year Bachelor’s phase and a 3-year Master’s phase. The Bachelor’s program includes 2 Dutch-taught and 2 English-taught tracks, called learning communities. The program is problem-based and patient- and student-centred, with a focus on tutor groups, practicals, and e-learning rather than lectures. The students are expected to be proactive, and they are encouraged to develop self-regulated and self-directed learning skills to pursue lifelong learning. The 3-year Master’s program includes 2.5 years of clinical rotations (1 year of junior clerkships, 1 year of senior clerkships, 0.5 year of elective clerkship), and 0.5 year master thesis.

The 6-year undergraduate dentistry curriculum of the University of Groningen, likewise, is comprised of a 3-year Bachelor’s phase and 3-year Master’s phase and has a patient-centred approach. Compared to medicine, the dentistry Bachelor’s phase has a stronger focus on lectures and practicals and is taught in Dutch only. The Master’s phase consists of 1 year of mainly skills labs and practicals, while the final 2 years mainly consist of clinical rotations and a master thesis. Both medicine and dentistry students use e-learning, and teachers sometimes apply GBL, but there is no considerable nor structural implementation of GBL in either curriculum.

### Participants and Ethical Considerations

We used convenience sampling and invited all medical and dental students to participate in our study via an online announcement on the virtual learning environment of the University of Groningen, which is also used as a communication platform and is visible to all students (N=1600). We explained that the purpose of the focus group study was to gain more insight into students’ perceptions of play due to increasing interest in GBL. It was communicated that students not interested in games would also be able to participate in this study. We did not set specific exclusion criteria.

Ethical approval was obtained via the Netherlands Association for Medical Education (January 11, 2019). Prior to the start of each focus group session, the participants signed an informed consent form and completed a brief demographic questionnaire. They were informed that participation was on a voluntary basis and that they had the right to withdraw at any time if they were not comfortable with the study. After each session, participants received a gift certificate of €10 (US $11.88) for their time and effort.

### Focus Group Sessions

The focus group sessions followed the guidelines as described by Krueger et al [42] as well as the Association for Medical Education in Europe (AMEE) guideline on using focus groups [41]. Initially, 6 focus groups sessions (4 Dutch and 2 English sessions, with a maximum capacity of 13 students per session) were planned, each lasting 1.5-2 hours.

With the consent of the students, all meetings were audiotaped for later transcription and analysis. It was explained that there were no correct nor incorrect answers and that we were interested in all ideas and perceptions. The discussions were structured around a short break. Before the break, discussions aimed at exploring playful behavior in leisure time. After the break, discussions continued and focused on participants’ ideas and perceptions of the play-learning interaction and how GBL could be implemented in the curricula to foster their learning. We used a topic list with open-ended questions (Textbox 1) and encouraged further discussion. The first 4 sessions were moderated by 1 of the authors (AJ). An observer (Ob1) was seated outside the group and took detailed field notes of group dynamics, atmosphere, and nonverbal communications. The last 2 sessions were moderated by the observer of the first 4 sessions and, consequently, a different observer (Ob2) was used. To create an open and social atmosphere, pizza and soft drinks were served.

After 4 sessions, our sample provided sufficient information power to address the aims of this study [43]. The information we had gathered from these focus groups was used to fine-tune and add some questions to the topic list for the final 2 focus group sessions (Textbox 2). Since no new information was obtained in these 2 sessions, we decided not to schedule any further sessions [42].

**General question route for focus group discussions.**

1. Opening question
  What is your favorite game?
2. Discussion on games and gameplay
  Why do you like your favorite game?
  Which type of games do you dislike? And why?
  How does your favorite game night look like?
  What do you think about when thinking of playing games?
  Do you play less now than when you were young? Why so? Do you wish it were different?
3. Break
4. Discussion on game-based learning and implementation
  Try to think about how you would like to use a game or game elements within the current education. What would that look like? Try to invent something in groups of 2 / 3 that you would actually like to use yourself.
  Let's talk about your ideas. Why did you choose this course and these game elements?
  Is your intrinsic motivation (not) enough obvious? Is using game-elements really necessary?
  Suppose you are the director of your education; how would you organize your education with GBL?
5. Summary
  “summary of discussion” Did I summarize it correctly? Anyone want to add something?

**Additional questions**

- What does a game make ‘addictive’?
- Does anyone not like to play games?
- When do you prefer to play?
- Do you ever play drinking games? Why do you think that is attractive?
- Which type of play elements do you believe would work in education?

### Data Analysis

All audiotapes were transcribed verbatim and anonymized before analysis. Atlas.ti (version: 8.4) was used as software to help us manage and analyze the data [44]. The method of thematic analysis was used to evaluate the data [42]. We used the most widely adopted approach for thematic analysis [45] outlined by Braun and Clark [46] and consisting of 6 steps: (1) familiarization with the data, (2) generating initial codes, (3) searching for themes, (4) reviewing themes, (5) defining and naming themes, (6) producing the manuscript. Notably, this method of analysis is recursive, meaning that each subsequent step in the analysis might have prompted us to circle back to earlier steps in light of newly emerged themes or data [45]. The detailed observers’ field notes facilitated additional exploration of themes when needed throughout the entire process.

First, coders (AvG and Ob1) familiarized themselves with the data by examining and re-examining the transcripts and audiotapes. Second, initial codes were generated (Avg and Ob1) to organize the data on potential items of interest [45]. One focus group discussion was coded (AvG and Ob1); thereafter, the coders discussed and defined a coding framework for the remaining dataset while denoting possible patterns or discrepancies between the codes (Table 1) [46]. All disagreements between coders were resolved via discussion between the coders. Open coding was used to ensure flexibility to incorporate themes outside our questioning route or initial coding table (Table 1) [47]. Third, the identified codes from all focus groups were discussed with the entire team in order to construct themes. We inductively [41] and iteratively constructed themes by comparing, analyzing, combining, and mapping codes [45]. Fourth, the team (iteratively) reviewed the identified themes to examine whether they were sufficiently common and coherent, but also whether they were sufficiently distinct from each other to justify separation [45,46,48]. Fifth, we ensured that the denominators of our themes were adequately clear and descriptive. Finally, we wrote the manuscript, which proved to be a continuation of the iterative interpretation and analytic process of thematic analysis [49].

**Table 1:** Initial coding framework.

Preliminary codes	Examples
Meaning	Luck and unpredictability, ownership, meaning
Escapism	Fantasy, immersion, escapism, relaxation
Social	Being together, helping each other
Strategy	Strategy
Mechanics	Duration, variation
Achievement	Challenge, wining, losing, competition, revenge, provocation
Devotion	Dark play, eagerness
Exploration	Storytelling, learning new things, curiosity
Applicability for learning	Difficulty subject, boring, paradox with leisure

### Reflections

Our research team consisted of researchers with various backgrounds, supporting a critical examination and interpretation of the data from multiple perspectives. During the team discussions, we deliberately addressed all these perspectives, while allowing each team member to make an equal contribution.

AvG has a medical degree, is appointed as a lecturer (ie, anatomist), has a research interest in the motivational pull of play and games, and develops GBL strategies. JG is an associate professor of anatomy with a research interest in affective neuroscience and motivational forces in education. AJ is a full professor of Health Professions Education and Research with ample experience in qualitative research. Ob1 is a master student in Dentistry, assists multiple (clinical) educators in developing e-learning, and helped perform this study as part of her graduation assignment.

AvG and JG did not join the focus group sessions because they might know the participating students; AJ did not know any of them. Ob1 knew some participants in 2 out of 4 focus group sessions she observed; however, these participants did not consider this to be a problem, and they felt free to speak their minds. When Ob1 acted as a moderator, she did not know any of the participants. 

## Results

A total of 58 participants volunteered to participate (41 women and 17 men; mean age 22.8 years, range 18-31 years). This sample was comprised of 30 Bachelor students in Medicine, 8 Master students in Medicine, 2 Bachelor students in Dentistry, and 18 Master students in Dentistry. Of the participants, 51 reported to be of Dutch nationality and 7 of a nationality other than Dutch (Brazilian, French, Israeli, Italian, Saudi Arabian, Romanian, South African). The number of participants joining each focus group ranged between 7 and 13 students. One focus group session only included dentistry students (n=13), 1 session was predominantly attended by dentistry students (6 of 7 were dentistry students), 1 session was predominantly attended by medicine students (11 of 12 were medical students), and 3 sessions (including the 2 English sessions) were only attended by medical students. We found no distinct differences between the opinion on play or GBL between dentistry and medical students. The detailed field notes yielded no additional results for analysis.

We chose to present our findings based on the structure of the focus group discussions: first, students’ perceptions of play in leisure time, then their perceptions of GBL, and finally, the interaction between play and learning. In the following sections, quotations are used to illustrate the findings. Identified themes are in ***bold and italic***, and identified sub-themes are **bold**.

### Perceptions of Play in Leisure Time

At the start of each focus group session, students discussed their favorite games in leisure time. A great diversity of favorite game genres was mentioned by the students, for example, puzzle/jigsaw games, shooting games, strategy games, sport games, and adventure games. As one student stated: “I think there’s no game that everyone likes to play…”

All students liked to play, but the amount of play in leisure time ranged considerably from only once a year to daily. ***Pleasure*** seemed to be the common ground as to why students engaged in games, in all their diversity.

### Pleasure

Whether the games were solitary (eg, patience, jigsaw, or shooter games) or multiplayer or collaborative (eg, Monopoly, settlers of Catan, or FIFA), students felt that games should be fun. However, ways to achieve a pleasurable experience from play varied considerably across students.

For instance, fun could come from the **joy of winning** (“I really like winning.”), from the **feeling of supremacy and achieving something** (“You are special. You have something that others don’t have.”), or from **the delight of getting a good story out of a game** (“I’ve always seen video games as ‘my book kind of thing.’ I don’t read a lot of books, so I get my stories from games.”)

A striking aspect that was highlighted in the discussions was that not only the pleasure experience itself (eg, the experience of a victory) but also the **sense of pleasurable anticipation** motivated students to continue playing: “I continue playing until I win the final match.”

Students indicated that pleasure should **not be easily obtainable:** “it has to be a challenge to win.”

Reward uncertainty seemed to modulate the impact of the pleasure experience, such that uncertain wins were associated with greater pleasure than certain wins. Upon analyzing the students’ statements, it became clear that they experienced greater joy after a difficult win, compared to easy wins:

… father always wins [at Scrabble], that’s not the worst. … it also gives more satisfaction if you beat him.

What I like, is when you really make a brilliant move, so someone else just doesn't win, but you do.

However, the degree of reward uncertainty seemed to have an optimum. Students said that if the reward seemed out of reach and their chances of winning were little to none, or even close to a certain loss, all anticipatory tension was gone. When students no longer had fun or prospect of pleasure, they felt less motivated to continue playing:

When you keep losing, you’re done with it

the game

It [Monopoly] takes too long. You’re like “let’s just stop, do we really have to finish [the game]?”

The final major part of the pleasure experienced in play that was brought up in the discussions was social pleasure. Students tended to play games in groups of close friends or family or with new people (met in pubs, societies, or a digital world), which helped them gain or strengthen the sense of collectiveness and sociability:

It [playing a game with each other] makes you feel like you are in your own world.

You can talk about the game and about everyday life, which offers opportunities for discussion.

… then you just want it [the game] to last a long time, because you have such a good time with each other.

Students mentioned that play more easily **creates a bond**, a sense of social togetherness, which in turn can also be enriched through play: “… it makes you feel connected.”

However, the sociability of play could also backfire when players with competitive spirits who could not win (sore losers) ruined the game:

I’m very fanatic. If I lose, I’ll also be grumpy for an hour. A lot of people also don't like to play a game with me.

The sociability of play could also backfire when players who disliked strong competition were disappointed because play was merely reduced to competitiveness and the desire to win:

If they are all very fanatic, it (the game) doesn’t matter that much to me anymore.

### Perceptions of Game-Based Learning

In order to keep the balance between play and the serious act of learning, participants brought up and discussed their ***perceived requirements*** and the ***relevance*** for implementing GBL.

#### Perceived Requirements

Despite a possible unpleasant confrontation with the serious world (see the Paradox section), competition was believed to enhance learning:

The more you compete, the more you will learn by yourself, because you want to improve.

However, in order to keep competition playful and in balance with the more serious part of learning, students felt that players’ identities should remain confidential in GBL using competition (for example by choosing a nickname) or that players should be grouped into (collaborative) teams:

They [the other students] are allowed to see the [game] results, but then anonymously.

When you’re losing [a teamplay game], you’re not losing alone. So that’s also nice.

Students’ perceptions of meaningful GBL design generally stayed **close to their learning task** at hand (ie, the learning task itself could be easily recognized). Students particularly referred to disciplines they found difficult or tedious, such as anatomy, physiology, cell biology, immunology, or statistics:

… if courses are really tedious and dry, it [GBL] shows you that it’s [the course content is] useful, and if you play it right, the [new] knowledge sticks.

…if you can find a game to make people understand physiology, you’re a genius!

Furthermore, students often mentioned **game versions of their future workplace** (based on The Sims game), which gave them opportunities to learn playfully by building their own practice:

I used to play The Sims a lot and really liked to build. … Wouldn’t it be great to build your own dentistry practice in a Sims kind of way!? Designing your practice, doing treatment, making money to go to courses in which you can learn new treatments, through which you can make even more money so you can improve your practice, can get more staff etc. …

#### Relevance

Students’ opinions on the need for GBL were **divided**:

I don't necessarily want to play a game every time I go to class.

… yes, I think we need it [GBL].

Although there was some debate about how frequently GBL should be used, the consensus seemed to be that GBL could support learning. At times, the medical education continuum was experience as long-lasting, hectic, and stressful:

It [dentistry] is really a study for the long haul.

We are all really stressed, and everyone’s stressing each other out, like: “Have you passed the exam/the test?” or “Did you hear? He hasn’t passed it [the exam/the test]!”

Subsequently, students mentioned that adding playful fun to learning might help **relieve stress** in stressful times:

Why not make it a little more light-hearted? Just to relieve the tension every now and then.

Nevertheless, students felt that there has to be a **balance** between the playful and the serious, which has to be respected:

If we turn aspects of the 6-year learning process into play, it feels as if the/all seriousness has been lost.

The extent to which the serious and the playful should be balanced depended very much on personal preferences. Therefore, an approach **tailored** to students’ needs would be the best fit according to the students:

Make it an extra activity, because playing a game just doesn’t work for some people…

I think it’s also important to keep in mind that everybody is different ...

### Perceptions of the Interaction Between Play and Learning

**Figure 1:**
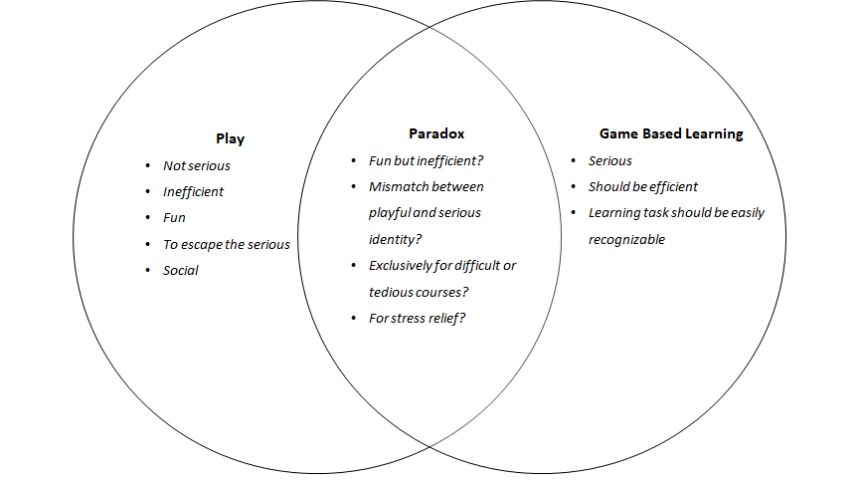
A paradoxical interaction between play and game-based learning based on identified subthemes.

The combination of play and the serious act of learning seemed ***paradoxical*** to students (Figure 1).

#### Paradox

Students overwhelmingly indicated that GBL should **not** be made **compulsory**. Compulsory play sounded for them like a “contradictio in terminis”: Play would become serious, which cannot be play:

If you are forced to play games, it would be like school.

If it [a GBL activity] becomes compulsory, then I don't like it anymore.

Students considered play to be a leisure-time activity to temporarily **escape** the serious demands of daily life:

It’s really relaxation, just something completely different [play], which has nothing to do with anything else.

Integrating play and the serious act of learning into education, therefore, seemed to be a paradox:

I think it’s strange that you can be enjoying gameplay in your private life for fun and relaxation, but apparently, if you frame it as “education,” it suddenly becomes too much.

Indeed, although students were apt to think of play as pleasure during leisure time, it was difficult for them to link play and pleasure to academic learning:

I find it difficult to see it [learning/GBL] as a game because it’s all so serious. Something is depending on it. And when I think of playing games, I think “Ah, cozy, fun!”

If I have to get together with everyone for a joint activity [GBL], then I think “No, I just have my own way of studying. And if I deviate from that, then I get really upset.”

Students believed that adding play [to education] would **reduce their learning efficiency**:

You probably have to “camouflage” the learning [part], which will probably require more study time.

I think (educational) games just have to be short and efficient…

If something [GBL] really takes a lot of time, then people are inclined to think, as always: “I just quickly read this [book] instead of wasting my time on a game.”

In addition to reduced learning efficiency, the paradox of play and learning was attributed to a **mismatch in identity** in play as compared to reality:

That’s the funny thing with games; you can pretend to be different than you normally are.

Losing a game in the imaginary play environment was never seen as fun but considered trivial nonetheless; from a gameplay point of view, the game was over, and the ending was (most of time) appreciated: “It’s just a game.”

However, students felt that their playful imaginary identity would be lost in learning. Losing a game in a learning activity or environment was considered to possibly lead to unpleasant and stressful confrontations with the real world:

… because it’s a game, just a one-time thing. And here, even if it’s just a Kahoot, and in general, sometimes you just have a group of questions you really don’t know anything about. But you can take it personally, even though you don’t have to. And think “I’m not a good student, but I want to become a good doctor” and “they’re all going to be better doctors than me” and “my resume is not good enough.”

In such cases, students particularly mentioned that competition had influenced this **unfavorable confrontation with the serious**:

I think you don’t want to show [your peers] that you’re not able to do something [well], and if you do it [GBL] in the form of a competition, that there’s always someone better than you. You have the feeling that you’re less good at it.

Or, students were concerned that competitive behavior in games would become prominent in their education as well:

I’m already chasing all the credits [in the curriculum] and I feel like it [competitive GBL] would make me too competitive, too reward focused.

## Discussion

In our study, we took inspiration from play research as a first step towards a mechanistic analysis of GBL effects. On the basis of open focus group discussions, we explored how medical and dental students perceived play in leisure time as well as in the context of academic learning, GBL in particular. The student samples were representative of the student population in terms of age, intellectual level (university students), and academic interest (medicine and dentistry). All students reported that they liked to play in leisure time. However, analysis of the transcripts showed that they had very different ideas about how pleasure could be achieved through play. Although we intentionally did not refer to a specific definition or conceptualization of play during the focus group discussions, students naturally discussed play in the context of digital, card, and board games. At the evaluative level, we observed a strong tendency towards rule-bound “ludic” play and only a weak tendency towards free, creative “paidic” forms of play [34,40,50-52]. An important observation from our analysis pertains to the context-dependency of the reported playfulness. Students openly and enthusiastically discussed play in leisure time, but when they were asked to discuss play in the context of GBL and academic learning, they began to carefully formulate their perceptions of play. They became cautious and began to change their perceptions of play, and many even became sceptical or disapproving. It seems that the outcomes of our focus group study not only allowed us to confirm some of the key principles of adult play (eg, challenge and sociability) [34,50,51,53] but also enabled us to generalize these to the context of health profession education. Moreover, we were able to identify key elements to consider in deciding if, how, and when to adopt GBL in health profession education to, possibly, foster learning.

Pleasure was a central theme in the open focus group discussions. This is not surprising because play, in its most fundamental expression, is seen as one of the primary positive emotions common to all mammals. Interestingly, students’ perceptions of what made play pleasurable varied considerably and involved not only positive affect (eg, fun, sociability) but also affective states that can be taken in a more negative way (eg, the urge to win). This variation persisted across participants and focus group sessions, even though the focus group composition was similar regarding demographic characteristics. This finding is consistent with previous literature stating that play preference, inclination to play, and the meaning of play are associated with many variables such as culture [54-56], personality [57-59], gender [60-62], and play frequency [63,64]. Attesting to the variable nature of play liking is that even negative affect, such as feelings of guilt or antisocial behaviors like sadism and violence [65-67], can be pursued in play and games and might be considered pleasurable in certain contexts [68-71]. It thus seems clear that in humans, any analysis of the interaction between pleasure and play must also exceed the level of primary emotion.

In our analysis of the transcripts, we adopted a multilevel conceptualization of pleasure [72], where we consider pleasure as more than just the joy of playing, which resonates findings from other fields such as developmental psychology, psychoanalysis, and neuroscience [18,72-74]. Pleasure research showed that various positive and negative behaviors and incentives can activate the same pleasure system in the brain and that pleasure is contextual and mainly dependent on individual experiences [75,76]. Pleasure can also refer to mood states (eg, happiness [77], a feeling of content), which can be maintained by perseverance [78,79], even at the cost of momentary negative affect [80,81]. Human play has also been associated with interest [82-84], surprise [85-87], and arousal [88-90]. In our focus group discussions, some students mentioned that the anticipatory joy of possibly winning as a main reason why playing was fun for them. Students also discussed the pleasure of uncertainty in this respect. On one hand, they perceived the pleasure of uncertainty (imagining winning the game) as more motivating than the pure pleasure of certainly winning (“difficult wins over easy wins”). On the other hand, they perceived a high probability of not being able to achieve the desired outcome (certain loss) as demotivating and in fact, as a reason to end the game, which is in line with the literature [91]. Indeed, is it well established that reward probability has a powerful effect on the anticipatory state of pleasure; the greater the reward uncertainty, the greater the motivating effect will be, but only if there is (at least) some probability in receiving that reward [91,92].

Another frequently discussed element of play pleasure was sociality. Many students believed that playing together was way more fun than playing alone. This is in line with findings of research in animals other than humans, in which social play is characterized as a high-level reward [93,94], more pleasurable than other forms of social interaction [95-97], and, intriguingly, at times even more pleasurable than food [98,99]. Also in human studies, it has empirically been shown that a prominent characteristic of social play is its high reward value [98,100]. However, some students appeared to be hesitant of social play; they perceived that pleasure gained from social play was sensitive to any dominance hierarchy within the player group.

A main observation from the focus group sessions was that students’ enthusiasm about play dampened when the context of the discussion shifted from leisure time to academic learning and GBL. Students mentioned many instances where play could be beneficial for their learning or even for their personal well-being. However, students also mentioned that GBL felt, at times, like a paradox and that play cannot be implemented in every course. Perhaps, this might be attributed to a shift from intrinsic motivation (free play in leisure time) and extrinsic motivation (when play becomes task-based and therefore, possibly, less fun). Another important aspect of this particular discussion was that students perceived the implementation of play as the opposite of efficient learning. This criticism had to do with the underlying belief that academic learning is serious and that the opposite of seriousness is play. Students indicated that the act of academic learning should be efficient but saw play as inefficient. Nevertheless, they seemed to justify the *inefficiency* of play when it could increase the *efficiency* of learning. They saw the greatest benefits for somewhat tedious, difficult subject matter. As a corollary, this may also imply that if students judge a particular learning activity as too playful, they will criticize it as inefficient and rather prefer to avoid participating. This is in line with the moderate enjoyment hypothesis theorizing that the general relationship between entertainment and learning is an inverse u-shape [101]. According to this hypothesis, entertainment (and the resulting pleasure) only facilitates learning up to a certain point, the peak of the inverted u-shape. After that point, learning will decrease, possibly due to distraction (leading to inefficiency) of entertainment [88]. Interestingly, but paradoxically enough, pleasure associated with playing may be perceived as hindering the achievement of higher (academic) goals, which, in turn, is also part of the pursuit of happiness and well-being by providing long-term pleasures [75].

Considering play as inefficient corresponds with the literature on this subject. For instance, Suits [51] stated that all play involves sacrifice of efficiency; there are always easier ways to obtain goals than through play. In golf, for example, there are far more efficient ways to get a small round object into the ground than with the swing of a golf club, but the voluntary acceptance of game rules permits the player to do so [51]. He and many others argued that, without the voluntary acceptance of these rules (with an inherent loss of efficiency), play will be lost [34,50-52]. Voluntarily accepting rules in favor of less efficient means also resonates with our finding. Students stated that play implemented as a learning tool (ie, GBL) should not become a compulsory activity for students. This is in line with work from developmental psychologists [102,103], research on motivational theories applied to play [104], and views from play scholars [34,50,51].

Students’ opinions about competition in play varied considerably, depending on the context. When students considered competition in the context of leisure time play, their focus was on the (prospective) joy of winning, and they could also interpret winning and losing as trivial outcomes of play. However, students saw it as a serious matter in the context of academic learning. They felt that competitive elements in a learning environment could possibly lead to unwanted and stressful confrontations with the serious world or make them too reward focused. Nevertheless, competition was believed to enhance learning but especially when played in teams or when played anonymously.

Finally, our findings can be explained by various theories on motivation and game design. For instance, the findings that students play for sociability and challenge, but need to feel free in doing so, closely ties in with the self-determination theory [105]. This theory has been linked to videogames [106], serious games [107], and gamification [108,109] in prior studies and states that individuals are intrinsically motivated when the basic psychological needs of feelings of competence (challenges), relatedness (sociability), and autonomy are met. Many of our findings reflect these psychological needs, yet students did not seem to prefer all 3 psychological needs simultaneously. For instance, some students implicitly mentioned that competence was an important indicator to continue play: the possibility to improve oneself. Others, however, did not play for competence, but rather for the sake of sociability (pertaining to the “relatedness” need). Also differing from the relatedness need was the finding of students who were interested in a single player game that draws them into the storyline. Autonomy on the other hand was very much agreed upon; play should not be compulsory. Building on the self-determination theory, Nicholson’s RECIPE for meaningful gamification is a design theory that describes 6 elements (Reflection, Engagement, Information, Choice, Exposition, and Play) in order to attain intrinsically motivated usage [109]. This theory is in line with many of our findings. For instance, GBL/play should be free (Play element), should be a choice (Choice element), should not deviate too much from the real-world setting (otherwise it might be deemed inefficient; Information and Exposition element), should be challenging and socially engaging (Engagement element), and should have a narrative (Exposition element).

### Practical and Research Implications

In research on GBL, design choices have rarely been made explicit, and most studies use the same type of design [4]. However, perceptions of play are highly individual, contextual, and variable, so one-design-fits-all approaches do not seem to work well for GBL research, according to our results. A thorough understanding of specific students’ perceptions within a culture or university might therefore play a pivotal role in utilizing the full potential of GBL. In the future, researchers and educators should map students’ play preferences before implementing GBL. Such information is essential for both the design of effective GBL activities and transfer of existing research into educational practice. As a first step in designing GBL and engaging in evidence-based decision making, teachers need to compare the play preferences of research participants in the study design with the play preferences of their own target group. This information also helps researchers understand and clarify what type of design works, for whom, and in what situation or circumstances. Thanks to the manageable and flexible possibilities of digital media, such an approach will bring tailor-made education a step closer.

Educators who want to implement GBL should aim at balancing the interaction between play and learning, harmonizing the right amount of play with the serious to increase efficiency of learning. What educators first should determine when they engage in teaching difficult subjects is whether there is a real need for play by identifying problems in students’ learning attitudes or learning behaviors [4]. Currently, determining the *right* amount of play and *how* learning efficiency can be improved seem to depend on intuition and personal perceptions rather than evidence-based decision and is therefore an area for future research.

One of our main findings was that participants strongly expected, or found, pleasure in play. The pleasure in games is strongly related to playing time [90]. Longer periods of time spent at GBL might indicate increased repetition of the learning material which, in turn, will lead to improved learning outcomes and retention [4,110-113]. Educators who want to design GBL could, therefore, adopt different positive motivational forces of pleasure as a method to guide their design. Using pleasure as a motivational force also opens up exciting new ways for research. Negative motivational forces (eg, violence) are also often observed in games [65,66], but their roles have rarely been investigated in the context of GBL [3,4,9,114].

We identified sociability as a major incentive for medical and dental students to play. Consequently, social play might be an interesting design option for GBL material. Strikingly, social play is underrepresented in GBL research, since most studies adopt a single player approach [4,9,26,114,115]. Although students felt that competition could enhance their learning, educators should be careful with implementing competitive elements in GBL, because these may also cause undesired effects such as increased stress. Playing in teams or in anonymity may be more appropriate options for such scenarios.

### Strengths and Limitations

A strength of our focus group study may be that it generated a rich understanding of students’ perceptions, experiences, and beliefs with respect to play and the interaction between play and learning. An experienced moderator guided the focus group sessions and stimulated in-depth discussions; we thoroughly explored students’ perceptions; 2 independent researchers identified codes; and the whole team, including the moderator, discussed and reflected on themes.

As in any focus group study, the identified themes unavoidably bear some relation to the original impetus for asking the questions and designing the interview guide. We tried to counteract this by actively encouraging input from all students during the sessions, even if it deviated from the original topic list.

In focus group studies, researchers sometimes meet with their participants to verify the generated themes. Although we did end every focus group discussion with a summary, to check whether our summary was appropriate to how the participants experienced it, we did not opt to meet with our participants, which potentially could have altered our outcome.

The gender ratio in our sample was imbalanced in favor of female students (70% were women). Although this ratio represents the Dutch medical student population [116], some countries might have gender ratios more balanced towards men (eg, medical schools in the United States have around 50% of their students as men [117]). Literature argues that the liking of play differs between genders [118,119]. Therefore, there is a possibility that our findings are more pertinent to female students. Yet, our aim was not to provide a generalization, or find a consensus, during the focus group. Even in our coding, we aimed to include varied opinions on play and GBL. However, although we tried to counteract an imbalance towards female students, results might be localized to the students of Dutch medical schools.

The meaning of play is associated with many variables such as culture [54-56]. Since we used convenience sampling, the participants in this study predominantly had a similar ethnic background (European/Caucasian) and possibly also a similar socioeconomic background [120]. Therefore, cultural and regional differences might have affected the results of our study. In a different setting, the same methodology might yield different results.

Finally, our findings on the play-learning interaction reflect students’ perceptions. Perceptions, however, do not always reflect actual behavior, meaning that students do not always do what they say they do. For example, in a study on using leader boards to increase the use of laparoscopic simulators, the majority of the surgical residents mentioned that they were not motivated by leader boards. However, the results showed that the time students in the competition group had spent on the simulation was higher than in the control group [121]. Additionally, because students in our sample had little experience with GBL, they might not have fully understood all the possibilities of GBL and, therefore, might have provided limited answers. Nonetheless, we believe that our findings offer important insights for future research to examine which design and GBL situation hold the highest promise for learning.

### Conclusion

With this focus group study, we aimed to explore students’ perceptions of play and the play-learning interaction. We explored what they considered to be play and how they believed it could interact with their learning. Four key points emerge from our study. First, students play for pleasure. Perceptions of pleasure vary considerably among students. Second, students consider play as inefficient. Inefficiency will only be justified when it increases learning. Third, play should be balanced with the serious and only used for difficult or tedious courses. Fourth, GBL activities should not be made compulsory for students, since there is a discrepancy between the serious of compulsory activities and the free nature of play.

## Abbreviations

AMEE: Association for Medical Education in Europe
GBL: game-based learning
